# Monocaprin Enhances Bioavailability of Fucoxanthin in Diabetic/Obese KK-*A^y^* Mice

**DOI:** 10.3390/md20070446

**Published:** 2022-07-07

**Authors:** Kodai Nagata, Naoki Takatani, Fumiaki Beppu, Aya Abe, Etsuko Tominaga, Tomohisa Fukuhara, Makoto Ozeki, Masashi Hosokawa

**Affiliations:** 1 Faculty of Fisheries Sciences, Hokkaido University, 3-1-1 Minato, Hakodate 041-8611, Hokkaido, Japan; ko.d12n@gmail.com (K.N.); n-takatani@fish.hokudai.ac.jp (N.T.); fbeppu@fish.hokudai.ac.jp (F.B.); 2 Taiyo Kagaku Co., Ltd., 1-3 Takaramachi, Yokkaichi 510-0844, Mie, Japan; aabe@taiyokagaku.co.jp (A.A.); etominaga@taiyokagaku.co.jp (E.T.); tfukuhara@taiyokagaku.co.jp (T.F.); mozeki@taiyokagaku.co.jp (M.O.)

**Keywords:** fucoxanthin, monocaprin, bioavailability, fucoxanthinol

## Abstract

Fucoxanthin is a marine carotenoid found in brown seaweeds and several microalgae. It has been reported that fucoxanthin has health benefits such as anti-obesity and anti-diabetic effects. To facilitate fucoxanthin applications in the food industry, it is important to improve its low bioavailability. We attempted the combined feeding of fucoxanthin-containing seaweed oil (SO) and monocaprin in a powder diet and analyzed the fucoxanthin metabolite contents in the liver, small intestine and serum of diabetic/obese KK-*A^y^* mice. After 4 weeks of feeding with the experimental diets, the serum fucoxanthinol concentrations of the mice fed 0.2% SO and 0.5% monocaprin were higher than those of the 0.2% SO-fed mice. Furthermore, fucoxanthinol accumulation in the liver and small intestine tended to increase in a combination diet of 0.2% SO and 0.125–0.5% monocaprin compared with a diet of 0.2% SO alone, although amarouciaxanthin A accumulation was not different among the 0.2% SO-fed groups. These results suggest that a combination of monocaprin with fucoxanthin-containing SO is an effective treatment for improving the bioavailability of fucoxanthin.

## 1. Introduction

Fucoxanthin is a marine carotenoid with unique structures such as an allene bond and epoxide in the molecule. It is an accessory pigment for photosynthesis contained in brown seaweeds and in several microalgae species. We have reported that dietary fucoxanthin exhibits beneficial functions to health such as anti-obesity and anti-diabetic effects in animal models [1,2]. Human studies have also shown a reduction in white adipose tissue weight and HbA1c level related to blood glucose regulation by fucoxanthin [3,4]. Therefore, there is interest towards fucoxanthin, for its utilization as a nutraceutical ingredient in the food industry. However, the uptake of fucoxanthin into intestinal cells is low compared with other carotenoids such as β-carotene and lutein [5]. Therefore, improving the low bioavailability of fucoxanthin is an important challenge.

In previous studies, lysophosphatidylcholine and lysoglycerogalactolipid has been reported to enhance carotenoid uptake into the intestinal Caco-2 cells [5,6]. These monoacyl-lipids are suggested to enhance the bioaccesibility of carotenoids, including fucoxanthin, by affecting the micellar state of digestive lipid components. In addition, fucoxanthin absorption was improved using an emulsion-based delivery system [7] and protein-based encapsulation [8]. Thus, improving the bioaccessibility and bioavailability of dietary fucoxanthin is important for enhancing its functionality.

Monoacylglycerol (MG) is an amphipathic lipid, and it is used as a nonionic emulsifier and as an additive in the food industry. The functional properties of MG depends on its fatty acid chain length and its unsaturation. Monocaprin, which is an MG-binding capric acid with a 10-carbon chain, is well known for its anti-bacterial activities [9]. A combination of monocaprin and doxycycline also showed effective anti-viral and wound-healing actions against herpes labialis [10]. Moreover, medium-chain fatty acids, including capric acid, are also reported to prevent the metabolic syndrome [11]. Thus, monocaprin is expected to be a functional lipid that affects the micellar state of carotenoids. Therefore, we paid attention to the ability of monocaprin to enhance the bioavailability of fucoxanthin. In addition, the mixing of fucoxanthin and monocaprin into a powder diet is a very easy-to-use method compared with delivery and encapsulation systems.

In this study, we investigated fucoxanthin bioavailability in diabetic/obese KK-*A^y^* mice via the combined feeding of fucoxanthin-containing seaweed oil (SO) and monocaprin.

## 2. Results and Discussion

**Figure 1:**
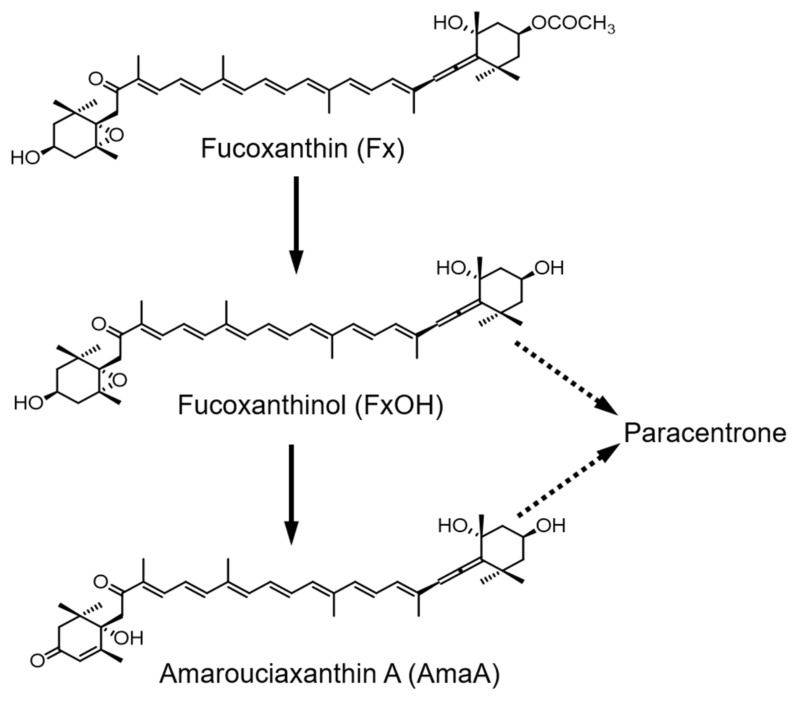
Fucoxanthin metabolites in the body.

Dietary fucoxanthin is hydrolyzed to fucoxanthinol in the gastrointestinal tract and is converted to amarouciaxanthin A in the liver (Figure 1) [12]. We also reported that fucoxanthin metabolites are transported and accumulate in several tissues, such as the liver, white adipose tissue, and skeletal muscle (Figure 1) [13,14]. To enhance the bioavailability of fucoxanthin, we attempted a combined feeding of monocaprin and fucoxanthin mixed in a powder diet on diabetic/obese KK-*A^y^* mice. In this study, we used SO containing 5% fucoxanthin and prepared the experimental diets as shown in Table 1. The 0.2% SO diet was prepared by replacing medium-chain triglyceride (MCT) in the control diet with SO (B, D, E, and F groups in Table 1). The fucoxanthin concentration was adjusted to 0.01% in the diet. Monocaprin was added into the diet at 0.125–0.5% by replacing soybean oil, as shown in Table 1.

After 4 weeks of feeding with the experimental diets and the control diet, there was no significant difference in the body weights among all of the groups (data not shown). SO-containing diets did not show an anti-obesity effect on KK-*A^y^* mice because the fucoxanthin content in the diet was 0.01%, which is low compared with that in previous studies [1,2].

**Figure 2:**
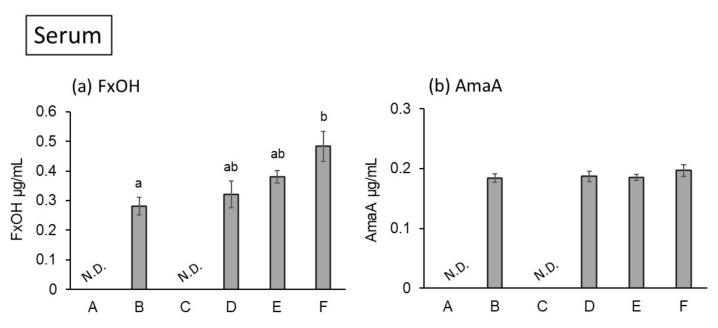
Carotenoid concentration in the serum of KK-*A^y^* mice fed experimental diets for 4 weeks. (A) Control, (B) SO 0.2%, (C) monocaprin 0.5%, (D) SO 0.2% + monocaprin 0.125%, (E) SO 0.2% + monocaprin 0.25%, (F) SO 0.2%+monocaprin 0.5%. (**a**) FxOH concentration, (**b**) AmaA concentration. SO: seaweed oil; FxOH: fucoxanthinol; AmaA: amarouciaxanthin A; N.D.: not detected. Bars with different letters are significantly different; *p* < 0.05.

Fucoxanthinol and amarouciaxanthin A, which are major metabolites of fucoxanthin, were analyzed, respectively, in the serum, and in the small intestines and livers of KK-*A^y^* mice. Fucoxanthin was not detected in all of the mice, as in previous studies [13,14]. In the mice fed a 0.2% SO diet, the fucoxanthinol concentration was 0.28 μg/mL (Figure 2a). The addition of monocaprin in the 0.2% SO diet increased the serum fucoxanthinol concentration in a dose-dependent manner. A combination of 0.2% SO and 0.5% monocaprin significantly enhanced the fucoxanthinol concentration at 0.48 μg/mL, compared with the 0.2% SO diet group (Figure 2a). On the other hand, the amarouciaxanthin A concentrations in the serum were not different among the SO-fed groups (Figure 2b).

**Figure 3:**
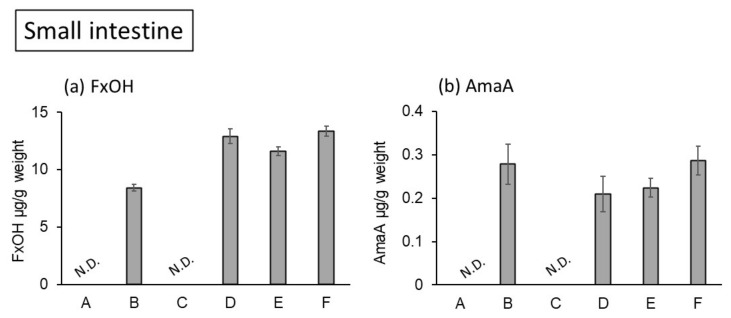
Carotenoid content in the small intestine of KK-*A^y^* mice fed experimental diets for 4 weeks. (A) Control, (B) SO 0.2%, (C) monocaprin 0.5%, (D) SO 0.2% + monocaprin 0.125%, (E) SO 0.2% + monocaprin 0.25%, (F) SO 0.2% + monocaprin 0.5%. (**a**) FxOH content, (**b**) AmaA content. SO: seaweed oil; FxOH: fucoxanthinol; AmaA: amarouciaxanthin A; N.D.: not detected.

**Figure 4:**
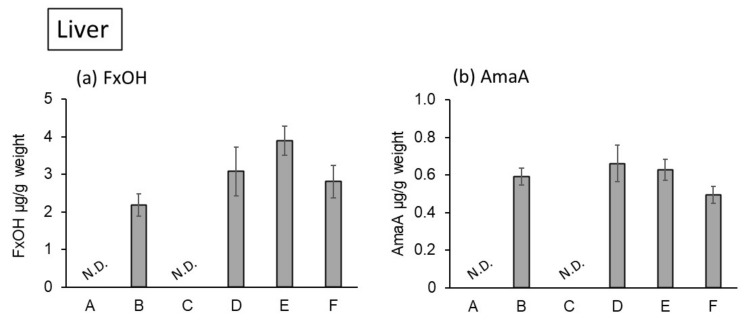
Carotenoid content in the liver of KK-*A^y^* mice fed experimental diets for 4 weeks. (A) Control, (B) SO 0.2%, (C) monocaprin 0.5%, (D) SO 0.2%+monocaprin 0.125%, (E) SO 0.2%+monocaprin 0.25%, (F) SO 0.2%+monocaprin 0.5%. (**a**) FxOH content, (**b**) AmaA content. SO: seaweed oil; FxOH: fucoxanthinol; AmaA: amarouciaxanthin A; N.D.: Not detected.

Fucoxanthinol accumulation was observed at 8.43 μg/g in the small intestine of the mice fed a 0.2% SO diet (Figure 3a). Monocaprin tended to increase fucoxanthinol accumulation in the small intestine but not in a dose-dependent manner. In the liver, fucoxanthinol accumulation also increased when using a combination diet of 0.2% SO and 0.125% or 0.25% monocaprin (Figure 4a). However, in the combination diet of 0.5% monocaprin and 0.2% SO, fucoxanthinol accumulation did not increase compared with that of the 0.2% SO and the 0.25% monocaprin diet. A high dose of monocaprin (0.5%) may activate the hepatic enzymes related to the degradation of fucoxanthin. This is required for further investigation.

On the other hand, amarouciaxanthin A accumulation was lower than fucoxanthinol accumulation in the small intestine and in the liver. Furthermore, amarouciaxanthin A accumulation in the small intestine and the liver was not different among SO-fed groups with/without monocaprin (Figure 3b and Figure 4b).

Fucoxanthin-loaded particles composed of casein and chitosan, or of albumin and oleic acid have been reported to improve fucoxanthin bioavailability 4 h or 24 h after oral administration to mice [15,16]. Fucoxanthin–oleic acid–albumin complexes that have been dispersed in water also improved the bioavailability and antioxidant capacity in the eyes as well as in the serum of mice, compared with free fucoxanthin, after 15 days of administration [8]. Furthermore, an emulsifier of fucoxanthin with gum arabic and γ-cyclodextrin dissolved in water was reported to improve the bioavailability of fucoxanthin [17]. These experiments were conducted via the oral administration of fucoxanthin complexes in the water system. On the other hand, we demonstrated for the first time that monocaprin together with fucoxanthin in a powder diet, but not in a water system, increases fucoxanthinol accumulation in the serum of mice after 4 weeks of feeding. These results show that a combination diet of monocaprin with fucoxanthin-containing oil is effective for improving the bioavailability of fucoxanthin. It is suggested that the addition of monocaprin in a fucoxanthin-containing diet may have an effect on the micellar state of carotenoids in the digestion system and may improve fucoxanthin bioavailability. Mixing SO and monocaprin into a powder sample is a very easy process. From the current results, it is expected that fucoxanthin will be applied widely within the food industry fields. 

We recently reported that dietary fucoxanthin inhibits hepatic oxidative stress and inflammation in non-alcoholic steatohepatitis model mice [18]. Therefore, our results provide beneficial information for the application of fucoxanthin and monocaprin in the food industry. On the other hand, the improvement of fucoxanthin stability is also important for applications. Sun et al. [19] reported that fucoxanthin microcapsules prepared using biopolymers are effective materials. Further examination is required to clarify the mechanism of a combined effect of fucoxanthin and monocaprin on stability as well as on bioavailability. 

## 3. Materials and Methods

### 3.1. Chemicals

SO (fucoxanthin-5KM composed of 79% food oil, 20% brown seaweed lipid and 1% tocopherol) containing 5% fucoxanthin was purchased from Oriza Oil & Fat Chemical Co., Ltd. (Aich, Ichinomiya, Japan). Monocaprin was prepared by Taiyo Kagaku Co., Ltd. (Mie, Yokkaichi, Japan). Other chemicals were obtained from FUJIFILM Wako Chemical Co. (Osaka, Japan).

### 3.2. Animal Experiments

The diabetic/obese KK-*A^y^* mice (4-week-old males) were obtained from CREA Japan Inc. (Tokyo, Japan). The mice were housed at 23 ± 2 °C and 50% humidity, with a 12 h light/12 h dark cycle and allowed free access to food and water. The control diet (A) containing 9.8% soybean oil and 0.2% MCT was prepared as shown in Table 1. MCT in the control diet was exchanged for 0.2% SO (B) in the experimental diets. Monocaprin was exchanged for soybean oil at 0.125–0.5% (C–F) in the experimental diets of each group, as shown in Table 1. After feeding the mice on a control diet for one week, six groups of seven mice were assigned so that there was no significant difference in body weight and blood glucose levels. Then, five experimental groups mice were acclimated by feeding a 0.5% monocaprin diet (C) for an additional one week. Control mice were fed a control diet (A), as shown in Table 1. After acclimation, each experimental diet and control diet (Table 1) were fed to the mice for 4 weeks. The mice were anatomized under anesthesia using isoflurane. The liver and small intestine were rapidly removed and stored at −30 °C. The serum was separated by centrifugation at 3000 rpm for 15 min. All procedures for animal care in this study were approved by the Ethical Committee of Experimental Animal Care of Hokkaido University (No 19-0083). 

### 3.3. HPLC Analysis of Fucoxanthin Metabolites

The total lipid (TL) was extracted from mouse tissues and serum with chloroform:methanol (2:1, *v/v*) containing α-tocopherol (50 µg/mL) according to the Folch method [20]. The obtained TL was dissolved in *n*-hexane:acetone (7:3, *v/v*) and subjected to an HPLC system (LC-20AD and CBM-20A (Shimadzu, Kyoto, Japan), column: Mightysil Si 60 250 × 4.6 mm (Kanto Chemical Co., Inc., Tokyo, Japan), two columns were connected, column temperature: 25 °C, mobile phase: *n*-hexane:acetone (7:3, *v/v*), flow rate: 1.0 mL min^−1^, detection: 450 nm). Fucoxanthinol and amarouciaxanthin A in the serum and tissues were quantified by HPLC analysis using the standard curves prepared by authentic standards. 

### 3.4. Statistical Analysis

The data are expressed as the means ± standard error of the mean (SEM). Statistical differences were determined via one-way ANOVA followed by the Tukey test at *p* < 0.05.

## Figures and Tables

**Table 1:** Compositions of control and experimental diets used in the animal experiment. Fx: fucoxanthin; MCT: medium chain triglyceride; SO: seaweed oil.

Ingredient (g/kg Diet)	A: Control	B: SO 0.2%	C: Monocaprin 0.5%	D: SO 0.2% +Monocaprin 0.125%	E: SO 0.2% +Monocaprin 0.25%	F: SO 0.2% +Monocaprin 0.5%
Soybean oil	98.000	98.000	93.000	96.750	95.500	93.000
Seaweed oil (SO)	-	2.000	-	2.000	2.000	2.000
(Fx in diet)	-	(0.100)	-	(0.100)	(0.100)	(0.100)
MCT	2.000	-	2.000	-	-	-
Monocaprin	-	-	5.000	1.250	2.500	5.000
Corn starch	374.120	374.120	374.120	374.120	374.120	374.120
Dextrinized cornstarch	124.240	124.240	124.240	124.240	124.240	124.240
Casein	207.000	207.000	207.000	207.000	207.000	207.000
Sucrose	94.120	94.120	94.120	94.120	94.120	94.120
Cellulose	50.000	50.000	50.000	50.000	50.000	50.000
AIN-93 mineral mixture	35.000	35.000	35.000	35.000	35.000	35.000
AIN-93 vitamin mixture	10.000	10.000	10.000	10.000	10.000	10.000
l-Cystine	3.000	3.000	3.000	3.000	3.000	3.000
Choline bitartrate	2.500	2.500	2.500	2.500	2.500	2.500
*tert*-Butyl hydroquinone	0.014	0.014	0.014	0.014	0.014	0.014

## Data Availability

Not applicable.

## References

[1] Maeda H., Hosokawa M., Sashima T., Funayama K., Miyashita K. (2005). Fucoxanthin from edible seaweed, Undaria pinnatifida, shows antiobesity effect through UCP1 expression in white adipose tissues. Biochem. Biophys. Res. Commun..

[2] Hosokawa M., Miyashita T., Nishikawa S., Emi S., Tsukui T., Beppu F., Okada T., Miyashita K. (2010). Fucoxanthin regulates adipocytokine mRNA expression in white adipose tissue of diabetic/obese KK-Ay mice. Arch. Biochem. Biophys..

[3] Abidov M., Ramazanov Z., Seifulla R., Grachev S. (2010). The effects of Xanthigen in the weight management of obese premenopausal women with non-alcoholic fatty liver disease and normal liver fat. Diabetes Obes. Metab..

[4] Mikami N., Hosokawa M., Miyashita K., Sohma H., Ito Y.M., Kokai Y. (2017). Reduction of HbA1c levels by fucoxanthin-enriched akamoku oil possibly involves the thrifty allele of uncoupling protein 1 (UCP1): A randomised controlled trial in normal-weight and obese Japanese adults. J. Nutr. Sci..

[5] Sugawara T., Kushiro M., Zhang H., Nara E., Ono H., Nagao A. (2001). Lysophosphatidylcholine enhances carotenoid uptake from mixed micelles by Caco-2 human intestinal cells. J. Nutr..

[6] Kotake-Nara E., Yonekura L., Nagao A. (2015). Lysoglyceroglycolipids improve the intestinal absorption of micellar fucoxanthin by Caco-2 cells. J. Oleo Sci..

[7] Ma Z.X., Khalid N., Shu G.F., Zhao Y.G., Kobayashi I., Neves M.A., Tuwo A., Nakajima M. (2019). Fucoxanthin-loaded oil-in-water emulsion-based delivery systems: Effects of natural emulsifiers on the formulation, stability, and bioaccessibility. ACS Omega.

[8] Liu Y.X., Qiao Z.C., Liu W.Q., Hou Z.Q., Zhang D., Huang L., Zhang Y.P. (2019). Oleic acid as a protein ligand improving intestinal absorption and ocular benefit of fucoxanthin in water through protein-based encapsulation. Food Funct..

[9] Thormar H., Hilmarsson H., Bergsson G. (2006). Stable concentrated emulsions of the 1-monoglyceride of capric acid (Monocaprin) with microbicidal activities against the food-borne bacteria Campylobacter jejuni, Salmonella spp., and Escherichia coli. Appl. Environ. Microbiol..

[10] Skulason S., Holbrook W.P., Thormar H., Gunnarsson G.B., Kristmundsdottir T. (2012). A study of the clinical activity of a gel combining monocaprin and doxycycline: A novel treatment for herpes labialis. K. Oral Pathol. Med..

[11] Nagao K., Yanagita T. (2010). Medium-chain fatty acids: Functional lipids for the prevention and treatment of the metabolic syndrome. Pharmacol. Res..

[12] Asai A., Sugawara T., Ono H., Nagao A. (2004). Biotransformation of fucoxanthinol into amarouciaxanthin A in mice and HepG2 cells: Formation and cytotoxicity of fucoxanthin metabolites. Drug Metab. Dispos..

[13] Airanthi M.K.W.A., Sasaki N., Iwasaki S., Baba N., Abe M., Hosokawa M., Miyashita K. (2011). Effect of brown seaweed lipids on fatty acid composition and lipid hydroperoxide levels of mouse liver. J. Agric. Food Chem..

[14] Takatani N., Taya D., Katsuki A., Beppu F., Yamano Y., Wada A., Miyashita K., Hosokawa M. (2021). Identification of paracentrone in fucoxanthin-fed mice and anti-inflammatory effect against lipopolysaccharide-stimulated macrophages and adipocytes. Mol. Food Nutr. Res..

[15] Koo S.Y., Mok I.K., Pan C.H., Kim S.M. (2016). Preparation of fucoxanthin-loaded nanoparticles composed of casein and chitosan with improved fucoxanthin bioavailability. J. Agric. Food Chem..

[16] Li D.H., Zhang Q., Huang L., Chen Z.H., Zou C., Ma Y., Cao M.J., Liu G.M., Liu Y.X., Wang Y.B. (2021). Fabricating hydrophilic particles with oleic acid and bovine serum albumin to improve the dispersibility and bioaccessibility of fucoxanthin in water. Food Hydrocoll..

[17] Kumagai K., Nebashi N., Muromachi A., Nakano Y., Ito Y., Nagasawa T. (2018). Emulsified fucoxanthin increases stability and absorption in rats. Nippon. Shokuhin Kagaku Kogaku Kaishi.

[18] Takatani N., Kono Y., Beppu F., Okamatsu-Ogura Y., Yamano Y., Miyashita K., Hosokawa M. (2020). Fucoxanthin inhibits hepatic oxidative stress, inflammation, and fibrosis in diet-induced nonalcoholic steatohepatitis model mice. Biochem. Biophys. Res. Commun..

[19] Sun X., Xu Y., Zhao L., Yan H., Wang S., Wang D. (2018). The stability and bioaccessibility of fucoxanthin in spray-dried microcapsules based on various biopolymers. RCS Adv..

[20] Folch J., Lees M., Stanley G.H.S. (1957). A simple method for the isolation and purification of total lipides from animal tissues. J. Biol. Chem..

